# Small Molecules Identified by an In Silico Docking Screen Targeting Anaphase-Promoting Complex/Cyclosome Subunit 1 (APC1) Potentiate Paclitaxel-Induced Breast Cancer Cell Death

**DOI:** 10.3390/molecules30040895

**Published:** 2025-02-14

**Authors:** Scott C. Schuyler, Rythm Gupta, Tran Thi Bao Nguyen, Cheng-Ye Weng, Hsin-Yu Chen

**Affiliations:** 1 Department of Biomedical Sciences, College of Medicine, Chang Gung University, Kwei-Shan, Taoyuan 333, Taiwan; ch7221489@iitd.ac.in (R.G.);; 2 Department of Otolaryngology—Head and Neck Surgery, Chang Gung Memorial Hospital, Taoyuan 333, Taiwan; 3 Department of Chemical Engineering, Indian Institute of Technology, Hauz Khas, New Delhi 110016, India

**Keywords:** cancer, cell division, anaphase-promoting complex/cyclosome (APC/C), anaphase-promoting complex subunit 1 (APC1), cell division cycle 20 (CDC20), docking

## Abstract

Delaying mitotic cell cycle progression has been proposed as a strategy to potentiate the effects of anti-mitotic anti-cancer drugs that induce multipolar mitotic spindles. Toward this end, we have performed an in silico docking screen targeting anaphase-promoting complex/cyclosome subunit 1 (APC1) at a conserved 10-amino acid surface site that was modeled to interact via a single hydrogen bond with the essential mitotic anaphase-promoting complex/cyclosome (APC/C) co-factor cell division cycle 20 (CDC20). Five molecules were identified after screening 15,000 small molecules. As a secondary in cellulo bioactivity screening, MDA-MB-231 genomically unstable aneuploid breast cancer cells were exposed to each compound in the absence and presence of 10 nM paclitaxel or 1 nM eribulin, the likely clinically relevant doses of these drugs in these cells. Two of the five compounds, which share a common 2-(trifluoromethyl)quinazolin-4-amine chemical structure, induced elevated levels of cell death in combination with paclitaxel, as observed by fluorescence-activated cell sorting (FACS). These two compounds will now serve as a starting point for further optimization and target validation experiments and for additional in silico screens in search of other chemically related small molecules that display more potent but specific anti-cancer cell effects.

## 1. Introduction

A class of successful anti-mitotic anti-cancer drugs including paclitaxel and eribulin has been proposed to execute their effects by targeting microtubules to promote the formation of multipolar mitotic spindles [1,2,3,4,5,6]. This is hypothesized to lead to increased rates of chromosome mis-segregation and to the formation of lagging chromosomes in the daughter cells without blocking mitotic progression and without by-passing the mitotic spindle checkpoint [1,2,3,4,5,6]. The increased levels of chromosome losses induced by these drugs are proposed to induce cellular stress responses, potentially via proteotoxic stress, caused by imbalances in protein expression levels within the highly aneuploid daughter cells, leading to increased levels of apoptosis/necrosis and/or senescence [5]. The lagging chromosomes these drugs induce are also proposed to promote activation of the cyclic GMP-AMP synthase-stimulator of interferon genes (cGAS-STING) pathway in the daughter cells, leading to leukocyte recruitment into solid tumors, which execute immunogenic cell death, leading to tumor regression [4]. Although this class of anti-cancer drugs is successful, they often fail to yield a positive clinical outcome, in part because of cytotoxicity in healthy human cells leading to limiting doses because of severe conditions such as neutropenia [7]. Therefore, one current goal in anti-cancer research is to try to identify molecules that can potentiate the effects of this class of successful drugs that induce multipolar mitotic spindles [6,8].

An increased level of multipolar spindles is also the proposed mechanism for the basis of the anti-cancer bioactivity of KIF18a inhibitors that are currently the subject of clinical trials including sovilnesib (AMG 650) (patent WO2021211549A1; clinical trial NCT06084416) and VLS-1488 (clinical trial NCT05902988). For example, it was observed that a decrease in APC/C-CDC20 activity, induced by CRISPR/Cas9 mutagenesis targeting APC/C subunits, led to a delay in anaphase entry that potentiated the activity of the KIF18a inhibitor AM-1882 [9]. Thus, the identification and/or development of molecules that can delay anaphase entry, potentially by targeting APC/C-CDC20, may have the ability to enhance the effectiveness of anti-cancer drugs such as paclitaxel, eribulin, vinorelbine or anti-cancer agents that are under clinical development, such as sovilnesib (AMG 650) or VLS-1488 [8].

We had previously analyzed the five highest-resolution structures of various APC/C-CDC20-containing complexes in the RCSB protein data bank (RCSB PDB) (accession numbers: 6Q6G; 6Q6H; 5G04; 5LCW; 6TLJ) and identified sites on the APC1 and APC5 subunits that may serve as potential target sites to disrupt the interaction between the APC/C holo-enzyme and the CDC20 co-factor [10,11,12,13,14]. We have also previously reported peptides derived from budding yeast Cdc20 that can suppress yeast APC/C-Cdc20 enzyme activity in vitro and delay mitotic progression in cellulo [15]. Peptides that contain regions of Cdc20 that are proposed to interact with Cdc23 (APC8B in humans) alone or Apc5 alone had more limited or no ability to inhibit yeast APC/C-Cdc20 activity in cellulo [15]. Among the peptides we screened, one of the strongest enzyme inhibitors was the PL29 peptide, which shares some homology within a region of human CDC20 that was modeled to interact with human APC1 [15]. Thus, this has prompted us to take the first steps here to search for small molecules that have a modeled ability to interact with the same potential CDC20-binding site on human APC1. Toward this end, we have performed an in silico docking screen that yielded five small molecules, two of which display the ability in cellulo to increase MDA-MB-231 breast cancer cell death in combination with paclitaxel.

## 2. Results

**Figure 1:**
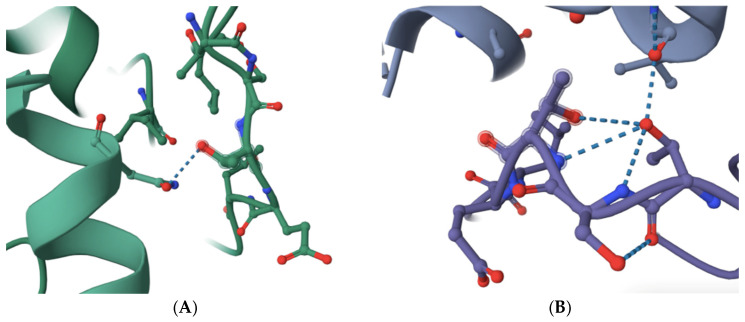
A comparison of the selected APC1 10-amino acid target site, 1236-LPPTSTELDV-1244, in the APC/C-CDC20-bound form and the APC/C-CDH1-bound form of APC1. (**A**) The modeled interaction between an N-terminal human CDC20 alpha-helix (left in green) and the APC1 target site (right in green) via a single hydrogen bond targeting threonine 1241 (dotted line) (RCSB PDB accession number 5G04). (**B**) The modeled interaction between a human CDH1 alpha-helix (top in purple) and the APC1 target site (bottom in purple) via a single hydrogen bond targeting threonine 1241 (dotted line) (RCSB PDB accession number 9GAW). Note the distortion of the APC1 target site in the CDH1-containing APC1 model through threonine 1241 forming additional hydrogen bonds with adjacent amino acid residues in the APC1 target site (bottom in purple).

The structure of budding yeast APC/C is very similar to that of human APC/C (see and compare, for example, RSCB PDB accession numbers 8A61 and 5G05) [10,11,12,13,14]. Based on sequence alignments and amino acid homology, the previously identified APC/C-Cdc20 inhibitor peptide PL29 target site may overlap with the site in human CDC20 that is modeled to bind with APC1 within a highly conserved 10-amino acid motif in yeast Apc1 (1110-LPSGSSDLNI-1119) and human APC1 (1236-LPPTSTELDV-1244) [10,11,12,13,14,15] (Figure 1). The number of modeled interactions in the N-terminal portion of human CDC20 between the C-box and the KILR-motif that are modeled to interact with APC/C subunits is very limited, to one potential interaction with APC1 through a single hydrogen bond and with APC5 via two potential hydrogen bonds [10,11] (Figure 1A). This contrasts with CDH1, which has extensive modeled interactions with APC1, including one potential hydrogen bond within the 1236-LPPTSTELDV-1244 motif and no modeled interactions with APC5 (see, for example, RCSB PDB accession number 9GAW) (Figure 1B) [10,11,12,13,14]. Furthermore, there is a notable distortion in the shape of the 1236-LPPTSTELDV-1244 motif in the CDH1-containing structure (compare the shapes in Figure 1A,B) where the critical target threonine 1241 folds back on the target site and is modeled to make several additional hydrogen bonds with adjacent amino acids in the target site (Figure 1B). Our reasoning was that if a small molecule can be identified that targets the 1236-LPPTSTELDV-1244 amino acids on APC1, CDC20 may be more sensitive to disruption of this interaction as compared to CDH1, an essential biochemical feature of any new molecule that might have the ability to delay anaphase entry, both because CDH1 has several other modeled interaction sites with APC1 that CDC20 was not modeled to have and because of the differences in the shape of the 1236-LPPTSTELDV-1244 target site in the two different APC/C isoforms that interact with different co-factors [10,11,12,13,14]. The 1236-LPPTSTELDV-1244 amino acids also have the added benefit that they are on the surface of APC1 and are in a location that is accessible because it is exposed to solution. The precise amino acid residue within the 1236-LPPTSTELDV-1244 target site that interacts with CDC20 remains unclear because different structures place the hydrogen bond between CDC20 and APC1 at different sites, but the most common interaction sites are within the three 1239-threonine–serine–threonine-1241 residues in the center of the selected target site.

**Figure 2:**
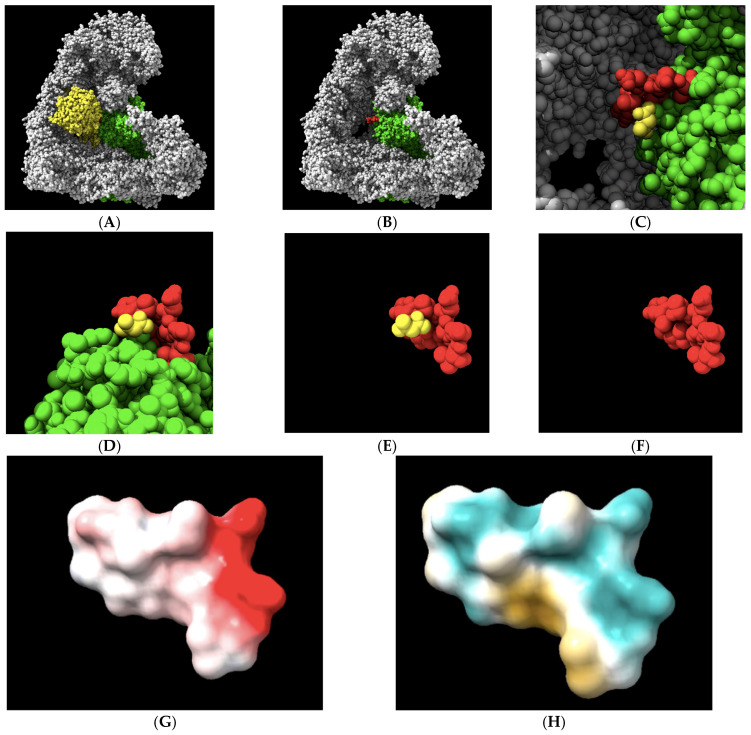
The geometrical and physical–chemical landscapes of the APC1 10-amino acid 1236-LPPTSTELDV-1244 target site (RCSB PDB accession number 5G04). (**A**,**B**) The APC/C (gray) holo-enzyme complex is visualized bound to CDC20 (yellow) or, in its absence, with the APC1 subunit of the complex labeled in green. The 10-amino acid target site on the surface of the APC1 is labeled in red. (**C**) The alanine from CDC20 that is modeled to interact with APC1 at the target site via a single hydrogen bond, displayed in yellow. (**D**–**F**) The 10-amino acid APC1 target site (red) displayed in a different orientation and in the absence of other APC/C subunits or in the absence of the remaining part of the APC1 (green), with the alanine from CDC20 that is modeled to interact with APC1 at the target site via a single hydrogen bond displayed in yellow. (**G**) The electrostatic potential landscape of the target site. Electronegativity is displayed in red, while electrically neutral regions are shown in white. The target, threonine 1241, is electrostatically neutral, while C-terminal aspartic acid 1243 displays the strongest potential, in red and toward the right. (**H**) The hydrophilic landscape, shown in aqua green, and the hydrophobic landscape, shown in yellow, of the target site reveal the bipartite nature of the target. The target, threonine 1241, is slightly polar in nature.

An initial analysis of the 1236-LPPTSTELDV-1244 target in an APC/C-CDC20 complex was performed to gain insights into its physical–chemical properties. We visualized the 10-amino acid target site in the context of the APC/C holo-enzyme (Figure 2A–C). We also visualized the 10-amino acid site in isolation to illustrate the central target location within the 10-amino acid motif that was modeled to interact with CDC20 (Figure 2D,F). Electrostatic potential analysis revealed a region along the upper surface of the target containing a negative charge with the potential to make an ionic bond or have a polar interaction toward the C-terminal region of the target (Figure 2G). However, the overall electrostatic potential was limited, with only a single charged residue on aspartic acid 1243 having a strong electrostatic potential. The most common locations of the CDC20 hydrogen bond with APC1 are with the three 1239-threonine–serine–threonine-1241 residues, which sit at the top of the landscape toward the left. An analysis of the distribution of the hydrophilic/hydrophobic regions on the target surface revealed a hydrophilic ridge across the upper region of the target that extended down one side, toward the N-terminal region (aqua-green color), and a separate hydrophobic pocket in the lower regions of the target, toward the C-terminal side (yellow–brown color) (Figure 2H). These features of the target landscape suggest that relatively small molecules with the proper three-dimensional shape to contour together within the slight curvature of the target space, containing polar and/or charged chemical moieties at one end and antipodal hydrophobic regions at the other end, may display the highest affinities for the target.

Based on this target-site physical–chemical landscape analysis, chemical libraries were pre-selected to enrich small-molecular-weight molecules that might have the ability to interact with polar groups and/or a negative charge as well as the hydrophobic motifs of a medium-sized molecular weight. In the end, 15,000 molecules were screened by docking using AutoDock Vina, and the top five molecules that displayed best-mode affinity binding energies between −6.0 and −6.4 kcal/mol were identified (Table 1) [16,17]. The molecules ranged in molecular weights between 347 and 435 Daltons.

**Figure 3:**
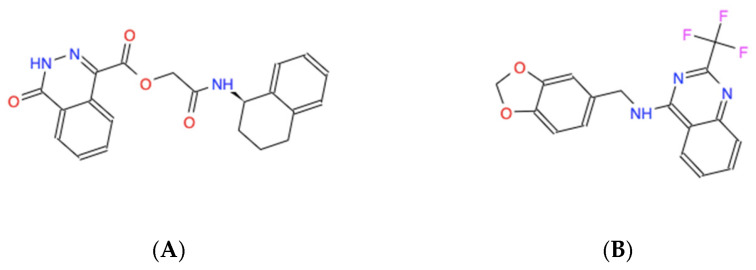

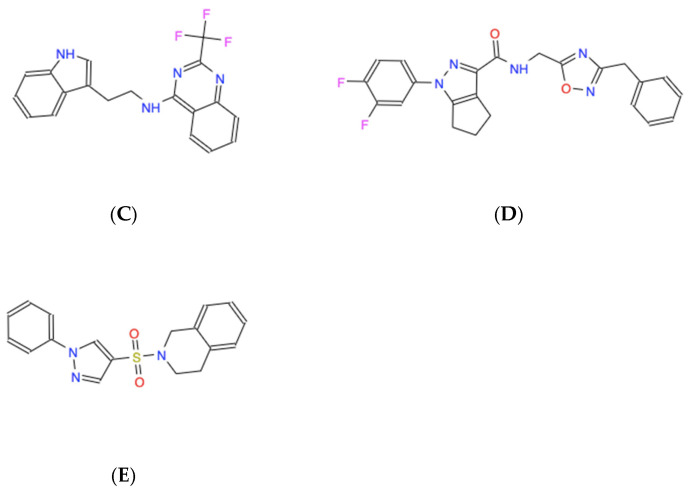
Skeletal chemical structures of the top five molecules: (**A**) C_21_H_19_N_3_O_4_ (ZINC000005182504) “408”, (**B**) C_17_H_12_F_3_N_3_O_2_ (ZINC000014197366) “558”, (**C**) C_19_H_15_F_3_N_4_ (ZINC000008038860) “734”, (**D**) C_23_H_19_F_2_N_5_O_2_ (ZINC000057991268) “164” and (**E**) C_18_H_17_N_3_O_2_S (ZINC000069037288) “686”.

The candidate molecules all share amphipathic structural elements, such as central polar chemical motifs containing nitrogen and/or oxygen combined with dual antipodal hydrophobic ring structures that likely facilitate the modeled interactions, as revealed by their skeletal structural formulas (Figure 3).

**Figure 4:**
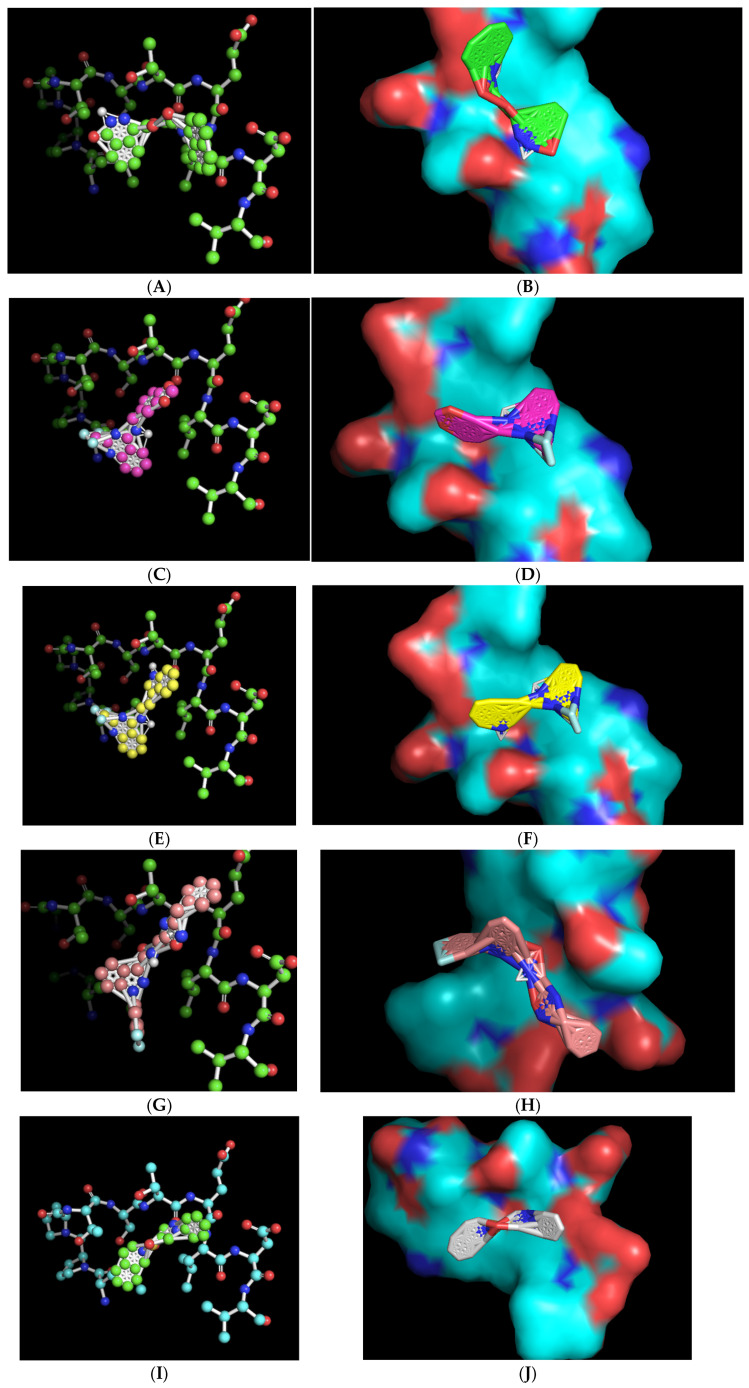
Ball-and-stick representations in similar orientations and three-dimensional contour models in various orientations of the best-mode affinity binding docked molecule geometries on the 10-amino acid APC1 target site. (**A**,**B**) C_21_H_19_N_3_O_4_ (ZINC000005182504) “408” orients with one portion of the molecule directly adjacent to and nearly on top of the target threonine 1241 (shown in red). (**C**,**D**) C_17_H_12_F_3_N_3_O_2_ (ZINC000014197366) “558” orients with one portion of the molecule adjacent to the target threonine 1241 (shown in red). (**E**,**F**) C_19_H_15_F_3_N_4_ (ZINC000008038860) “734” orients with one portion of the molecule adjacent to the target threonine 1241 (shown in red). (**G**,**H**) C_23_H_19_F_2_N_5_O_2_ (ZINC000057991268) “164” orients with the central portion of the molecule adjacent to the target threonine 1241 (shown in red). (**I**,**J**) C_18_H_17_N_3_O_2_S (ZINC000069037288) “686” orients in a manner much more distant from the target threonine 1241.

The ball-and-stick and three-dimensional models of the docked molecules on the 10-amino acid APC1 target site revealed a disparate array of interaction geometries (Figure 4). The first listed molecule, with chemical formula C_21_H_19_N_3_O_4_ (ZINC000005182504) “408”, displayed an optimized binding orientation that was somewhat parallel with the primary peptide backbone of the APC1 target site and had the added potential benefit of being in very close association with the target-site threonine 1241 amino acid residue, one of the common sites of the modeled interaction between CDC20 and APC1 (Figure 4A,B). By contrast, the molecules with formulas C_17_H_12_F_3_N_3_O_2_ (ZINC000014197366) “558” and C_19_H_15_F_3_N_4_ (ZINC000008038860) “734”, which share very similar overall chemical structures, displayed a vertical optimized association with the target site that was perpendicular to the peptide backbone but were also within a relatively close association of the target, threonine 1241, within the modeled CDC20 APC1 interaction site (Figure 4C–F). These two molecules also contain a tri-fluoromethyl motif that likely aids binding to the target site via hydrophobic interactions. The C_23_H_19_F_2_N_5_O_2_ (ZINC000057991268) “164” molecule also displayed a perpendicular vertical optimized interaction with the target site that featured a di-fluoro-containing ring at the base of the molecule but is of a different chemical nature and may have a slight disadvantage in that its association with the target site is more distant from the target threonine 1241 (Figure 4G,H). The final chemical, C_18_H_17_N_3_O_2_S (ZINC000069037288) “686”, has a unique central sulfonyl structure as compared with the other four molecules, and although it displayed an equivalent binding energy, its association was more distant from the target threonine 1241 (Figure 4I,J). All the three-dimensional structures revealed a close-fitting complementary contour between the slightly concave 10-amino acid APC1 target surface and the associated small molecules that appears to be facilitated by flexibility in the central regions of the molecules (Figure 4).

We obtained the five molecules at a purity of 90% and, for ease of use in the laboratory, gave them informal three-digit names based on the last three digits of their catalog numbers (Table 1) (Figure S1). The molecules were dissolved in 100% dimethyl sulfoxide (DMSO) at room temperature, which yielded colored or clear solutions that were either well-dissolved, in the cases of 558 and 734, or, with 408, 164 and 686, showing a small amount of a precipitate that remained undissolved in the tubes that was also visualized when these compounds were added into the cell culture media (Figure S2).

**Figure 5:**
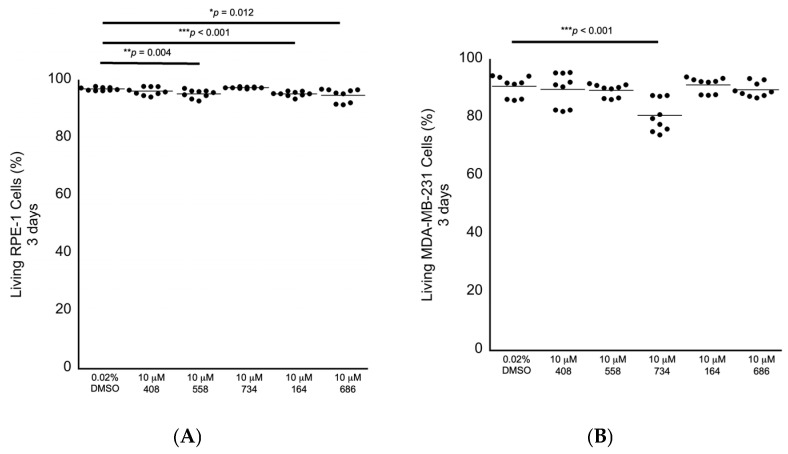
Cytotoxicity screening for 3 days as investigated by FACS analysis. (**A**) RPE-1 genomically stable diploid control cells displayed a small level of non-specific cytotoxicity in response to being exposed to 10 μM 558, 164 or 686. (**B**) MDA-MB-231 genomically unstable aneuploid breast cancer cells displayed a sensitivity to 10 μM 734 after being exposed for 3 days. All samples: n = 3 biological replicates; n = 9 technical replicates; one-way ANOVA; * *p* < 0.05; ** *p* < 0.01; *** *p* < 0.001.

We next performed secondary in cellulo screening tests for non-specific cytotoxicity by exposing genomically stable diploid RPE-1 retina epithelial cells, which are commonly used as a representative control cell line, to 10 μM of each compound for 3 days, followed by fluorescence-activated cell sorting (FACS) analyses to measure the levels of early and late apoptosis and the levels of necrosis as observed by annexin V labeling and propidium iodide staining. We chose 10 μM as the starting concentration for testing for bioactivity, as we have identified other molecules in our lab using a different approach that can enhance anti-cancer drugs at this concentration as a benchmark. RPE-1 cells exposed to each compound revealed that the small molecules 558, 164 and 686 displayed slight increased levels of non-specific cytotoxicity in the range of 1.8% to 2.2% relative lethality (Figure 5A; Table 2). We then exposed genomically unstable aneuploid MDA-MB-231 breast cancer cells to each of the five compounds individually at 10 μM for 3 days to investigate any potential anti-cancer cell cytotoxicity. The small molecule 734 displayed a cancer cell-specific cytotoxicity in the MDA-MB-231 cells, with an elevated relative lethality of 12.8% (Figure 5B; Table 2).

**Figure 6:**
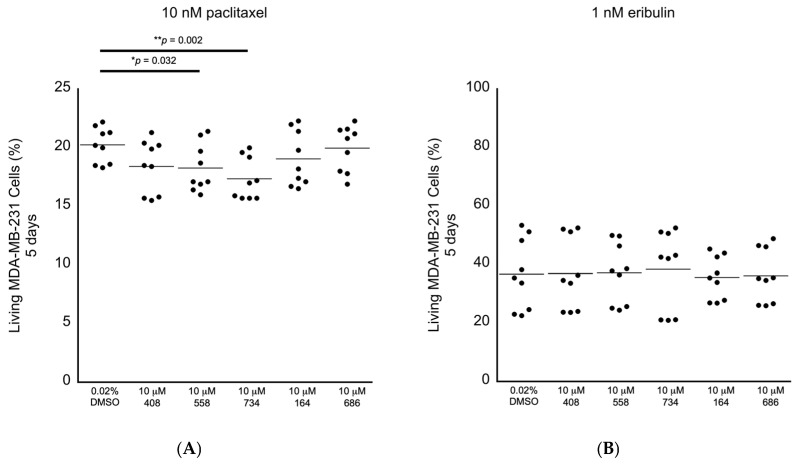
Screening for anti-cancer cell bioactivity in MDA-MB-231 breast cancer cells in combination with drugs that are proposed to act via increasing the levels of multipolar mitotic spindles. (**A**) Living MDA-MB-231 cancer cells in combination with the clinically relevant dose of 10 nM paclitaxel, where the small molecules 558 and 734 displayed significant differences compared to the 0.02% DMSO control. (**B**) Living MDA-MB-231 cancer cells in combination with the likely clinically relevant dose of 1 nM eribulin, where none of the small molecules displayed significant differences compared to the 0.02% DMSO control. All samples: n = 3 biological replicates; n = 9 technical replicates; one-way ANOVA; * *p* < 0.05; ** *p* < 0.01.

Having established the levels of cytotoxicity for each of the small molecules alone, we began screening the small molecules for the ability to potentiate the anti-cancer cell effects of chemotherapeutic drugs applied at the clinically relevant doses in the MDA-MB-231 breast cancer cells. We selected paclitaxel and eribulin because they are Food and Drug Administration (FDA)-approved drugs to treat breast cancer and because they are proposed to execute their anti-cancer cell activity by promoting multipolar mitotic spindles [1,2,3,4,5,6,8]. It has been established that 10 nM paclitaxel is the clinically relevant dose to use in MDA-MB-231 cells [1]. It has also been observed that 1–2 nM eribulin is the likely clinically relevant dose to use in MDA-MB-231 cells [6]. In this tertiary bioactivity screening, we observed that the small molecules 558 and 734 potentiated the induction of paclitaxel-dependent cell death at the relative levels of 9.8% and 14.4%, respectively (Figure 6A; Table 2). However, none of the five molecules displayed any ability to potentiate the effects of 1 nM eribulin on cell viability (Figure 6B; Table 2). The results of these in cellulo screening tests suggest that the small molecules 558 and 734, which are chemically similar via containing a common 2-(trifluoromethyl)quinazolin-4-amine chemical structure (see Figure 3A,B), are the best candidates to pursue for further optimization and validation amongst the five lead compounds.

## 3. Discussion

To our knowledge, no previous small-molecule screens in silico or in cellulo targeting the modeled CDC20 binding motif on human APC1 have been reported. There has also been speculation that this site may function as a target location for an APC1 auto-regulatory inhibitor loop [18]. We chose to target this specific site for three primary reasons based on our previous work and analyses: (1) in budding yeast, we had observed that the PL29 peptide displayed a robust ability to inhibit APC/C-Cdc20 activity in vitro, potentially by targeting the Cdc20-APC/C interaction site [15]; (2) because the three-dimensional structure of the APC/C holo-enzyme and the 10-amino acid target site in APC1 are highly conserved between the yeast (1110-LPSGSSDLNI-1119) and human varieties (1236-LPPTSTELDV-1244) [10,11,12,13,14]; and (3) because CDC20 is modeled to have only a single hydrogen bond-based interaction with APC1, which a small molecule might have the ability to disrupt, which is in contrast with the modeled interaction between CDH1 and APC1, which contains several modeled interaction sites, making it less likely that a single small molecule could disrupt the interaction [10,11,12,13,14]. The five novel small molecules identified here provided us with a starting point to explore targeting of the APC/C further. To our knowledge, none of these molecules has previously been tested in vitro as targeting the APC/C, and we have not identified any known bioactivity displayed by any of them in any previous publication. In addition, all five molecules are readily available in chemical libraries [16,17].

Our in cellulo secondary and tertiary screening validation studies revealed that 558 and 734, which both dissolved well in DMSO and both share a common 2-(trifluoromethyl)quinazolin-4-amine chemical structural element, have the best potential for further development. The small molecules 408, 164 and 686 were not fully soluble in DMSO at room temperature and they did not display any ability to promote cancer cell death, extinguishing their potential as candidate molecules. By contrast, the small molecule 734 was notable for promoting cell death in the MDA-MB-231 breast cancer cells on its own and in the presence of the clinically relevant dose of 10 nM paclitaxel, without any evidence of non-specific cytotoxicity in RPE-1 cells. It also appears that this anti-cancer cell cytotoxicity can be suppressed by 1 nM eribulin, which is an anti-microtubule drug that promotes microtubule depolymerization. This may imply that the observed cytotoxicity of 734 requires the presence of microtubules in the MDA-MB-231 cells. About 18% of MDA-MB-231 cells display multipolar spindles even in the absence of any drug treatment [3]. One speculative hypothesis is that these cells within the MDA-MB-231 population may be more sensitive to exposure to the small molecule 734. We intend to explore the bioactivity of the small molecule 734 further in other cancer cell lines, as well as in the presence of other anti-cancer microtubule depolymerizing drugs, such as vinorelbine, to investigate if its ability to promote cancer cell death is specific to MDA-MB-231 cells or if this molecule displays any broader level of anti-cancer cell activity. Finally, the small molecule 558 displayed a mixed bioactivity: it induced a small increase in non-specific cytotoxicity in the RPE-1 cells, but it also displayed the ability to potentiate the anti-cancer cell effect of 10 nM paclitaxel by promoting cell death.

Although the effects of molecules 558 and 734 are relatively small, the discovery of these molecular bioactivities provides a proof of concept in support of our in silico screening approach targeting APC1, and these two chemically similar molecules can serve as a basis for further optimization. In this regard, it may be notable that several current FDA-approved anti-cancer drugs with a variety of targets contain the 2-(trifluoromethyl)quinazoline-4-amine chemical motif [19,20]. In addition, several other N-aryl-2-(trifluoromethyl)quinazoline-4-amine derivative chemical compounds were also recently reported to display anti-cancer cell activity [19,20]. These studies did not include analysis of genomically stable diploid cell lines exposed to the compounds, but they did report intriguing bioactivities of various N-aryl-2-(trifluoromethyl)quinazoline-4-amine derivative molecules in the range of 11 nM to about 21 μM against PC3 (prostate cancer), K562 (leukemia), HeLa (cervical cancer), HEL (erythroleukemia) and LNCaP (prostate cancer) cells, targeting tubulin and the Werner helicase, which merits further study [19,20]. Using our discovery of the bioactivities of the small molecules 558 and 734, we intend to pursue optimization and target validation experiments and to perform additional in silico screens in search of other chemically related small molecules that display more potent but specific anti-cancer cell effects.

## 4. Materials and Methods

### 4.1. Protein and Ligand Data

For the source of protein and ligand data, the three-dimensional structure of the APC1 protein was obtained from the RCSB protein database with the accession number 5G04 (3.9 Å resolution) [10,11,12,13,14]. The target 10-amino acid sequence (1236-LPPTSTELDV-1244) on the surface of the APC1 was identified within the protein structure for further analysis, as described in detail in the Results section [10,11,12,13,14], and ligand libraries were collected from the lifechemicals database and were initially stored in a variety of file formats.

### 4.2. Molecular Visualization and Analysis

For the molecular visualization and analyses, the software ChimeraX (version 1.8) was used (University of California at San Francisco, CA, USA) [21]. The target motif (1236-LPPTSTELDV-1244) was visualized in the context of the APC1 protein structure using ChimeraX. For structural analysis, the target motif’s spatial arrangement and potential binding sites were observed using ChimeraX to examine the structural details of the amino acid residues and identify cavities or pockets that could be suitable for ligand binding. To perform electrostatic potential analysis, ChimeraX’s plugin functionalities, if available, were used to assess favorable electrostatic interactions for candidate drug molecules.

### 4.3. Chemical Format Conversion and Ligand Preparation

Open Babel (version 2.4.1) was employed for chemical data processing [22]. Ligand files in various formats were converted to AutoDock Vina-compatible formats (PDBQT) using Open Babel. Ligand structures were prepared through Open Babel by performing chirality checks, atom typing and generating 3D structures from SMILES strings as needed. This ensured compatibility with the docking software and accuracy in the simulations.

### 4.4. Docking Simulations

AutoDock Vina (version 1.2.4), released in May 2024 (The Scripps Institute, University of California at San Diego, CA, USA), was used for the docking simulations, and 15,000 molecules have been screened [23,24,25]. The grid box was centered at coordinates (179.072, 212.389, 178.848), with dimensions of 16 Å (x-direction), 24 Å (y-direction) and 20 Å (z-direction). The docking protocol was executed with an exhaustiveness level of 4, which controlled the thoroughness of the search. The decision to use an exhaustiveness value of 4 was guided by preliminary docking simulations, which demonstrated that further increasing exhaustiveness beyond this threshold yielded only marginal improvements in docking scores while substantially increasing computational time. To ensure an efficient screening process without compromising the reliability of the docking results, an exhaustiveness value of 4 was deemed appropriate. The docking results were analyzed to identify promising ligand candidates that interact with the target motif on the APC1 protein surface with the lowest calculated binding energies. The top five molecular candidates have the following ZINC database identity numbers and can be found here: <https://zinc.docking.org/substances/ZINC000005182504/, accessed on 12 February 2025>, <https://zinc.docking.org/substances/ZINC000014197366/, accessed on 12 February 2025>, <https://zinc.docking.org/substances/ZINC000008038860/, accessed on 12 February 2025>, <https://zinc.docking.org/substances/ZINC000057991268/, accessed on 12 February 2025> and <https://zinc.docking.org/substances/ZINC000069037288/, accessed on 12 February 2025> [14,15].

### 4.5. Culturing RPE-1 and MDA-MB-231 Cells and Exposing Them to Drugs/Small Molecules and FACS Analyses

RPE-1 hTERT-immortalized retinal cells (kind gift of B. Weaver, University of Wisconsin Medical Center) were cultured in Dulbecco’s Modified Eagle’s Medium (DMEM): F12 Medium (Gibco, cat: 11330-32, lot: 2906320), 10% fetal bovine serum (FBS) (Gibco, cat: 10437-028, lot: 2185285, Thermo Fisher Scientific, Waltham, MA, USA) supplemented with 100 units/mL penicillin-streptomycin (Gibco, cat: 15140122). MDA-MB-231 breast cancer cells (ATCC, HTB26, lot: 70049160, Manassas, VA, USA) were cultured in DMEM (Gibco, cat: 11965092) with 10% FBS and 100 units/mL penicillin-streptomycin (Gibco, cat: 15140122). The cells were exposed to 10 nM paclitaxel (Sigma, cat: T7402, St. Louis, MO, USA) or 1 nM eribulin (MedChemExpress, cat: HY-13442A, Monmouth Junction, NJ, USA) that was dissolved in dimethyl sulfoxide (DMSO) (Sigma, cat: D2650). The final amount of DMSO under each condition was 0.02% (*v*/*v*). Validation of the purity and chemical molecular identity for each of the small molecules was provided upon request from the manufacturer in the form of high-performance liquid chromatography (HPLC, Thermo Fisher Scientific, Waltham, MA, USA) and mass spectrometry spectra, with each displaying a prominent single peak (Enamine Ltd., Kyiv, Ukraine, cat: Z86108408; Z86226558; Z86252734; Z872761164; Z873176686) (Figure S1). The five candidate small molecules were dissolved in 100% DMSO as 50 mM stock solutions (Figure S2). FACS analyses were performed according to the manufacturer’s protocol (ThermoFisher, Attune NxT; Attune Cytometric Software, version 3.2.1) using propidium iodide (Sigma, cat: P4170) and Alexa Fluor 488-annexin V (Invitrogen, cat: A13201, lot: 2836690, Waltham, MA, USA) or Pacific Blue-annexin V (Invitrogen, cat: A35122, lot: 2647626).

## Figures and Tables

**Table 1:** The top five molecules identified by the in silico docking screen.

Chemical Name	Chemical Formula	Molecular Weight (Da)	Modeled Binding Energy (kcal/mol)	Compound Database ID (Catalog Number), Experimentally Referred to as “Name”
[(1,2,3,4-tetrahydronaphthalen-1-yl)carbamoyl]methyl 4-oxo-3,4-dihydrophthalazine-1-carboxylate	C_21_H_19_N_3_O_4_	377.39	−6.3	ZINC000005182504 (Z86108408) “408”
N-[(2H-1,3-benzodioxol-5-yl)methyl]-2-(trifluoromethyl)quinazolin-4-amine	C_17_H_12_F_3_N_3_O_2_	347.29	−6.3	ZINC000014197366 (Z86226558) “558”
N-[2-(1H-indol-3-yl)ethyl]-2-(trifluoromethyl)quinazolin-4-amine	C_19_H_15_F_3_N_4_	356.34	−6.3	ZINC000008038860 (Z86252734) “734”
N-[(3-benzyl-1,2,4-oxadiazol-5-yl)methyl]-1-(3,4-difluorophenyl)-1H,4H,5H,6H-cyclopenta[c]pyrazole-3-carboxamide	C_23_H_19_F_2_N_5_O_2_	435.43	−6.4	ZINC000057991268 (Z872761164) “164”
2-[(1-phenyl-1H-pyrazol-4-yl)sulfonyl]-1,2,3,4-tetrahydroisoquinoline	C_18_H_17_N_3_O_2_S	339.41	−6.0	ZINC000069037288 (Z873176686) “686”

**Table 2:** The relative absolute fold decrease in the mean cellular viabilities (%) for each small molecule normalized to the DMSO-only control.

Small-Molecule Molarity	Relative Fold Decrease in the Mean Value of Living RPE-1 Cells (%) at 3 Days in 0.02% DMSO	Relative Fold Decrease in the Mean Value of Living MDA-MB-231Cells (%) at 3 Days in 0.02% DMSO	Relative Fold Decrease in the Mean Value of Living MDA-MB-231 cells (%) at 5 Days in 10 nM Paclitaxel	Relative Fold Decrease in the Mean Value of Living MDA-MB-231 Cells (%) at 5 Days in 1 nM Eribulin
10 μM 408	-	-	-	-
10 μM 558	1.8%	-	9.8%	-
10 μM 734	-	12.8%	14.4%	-
10 μM 164	1.8%	-	-	-
10 μM 686	2.2%	-	-	-

## Data Availability

The original contributions presented in this study are included in the article/Supplementary Materials. Further inquiries can be directed to the corresponding author.

## Supplementary Materials

The following supporting information can be downloaded at: https://www.mdpi.com/article/10.3390/molecules30040895/s1, Figure S1. Validation of purity and chemical molecular identity by mass for each of the small molecules was provided upon request from the manufacturer in the form of high-performance liquid chromatography (HPLC) and mass spectrometry spectra, with each displaying a prominent single peak (Enamine Ltd., Kyiv, Ukraine). (A) C21H19N3O4 (ZINC000005182504) “408”. (B) C17H12F3N3O2 (ZINC000014197366) “558”. (C) C19H15F3N4 (ZINC000008038860) “734”. (D) C23H19F2N5O2 (ZINC000057991268) “164”. (E) C18H17N3O2S (ZINC000069037288) “686”. Figure S2. The top 5 candidate molecules dissolved in 100% DMSO at a final molar concentration of 50 mM as stock solutions.
