# Supplementary material for: Small Molecules Identified by an In Silico Docking Screen Targeting Anaphase-Promoting Complex/Cyclosome Subunit 1 (APC1) Potentiate Paclitaxel-Induced Breast Cancer Cell Death
Source: Molecules. 2025 Feb 14;30(4):895. doi: 10.3390/molecules30040895 (PMC11857951; doi:10.3390/molecules30040895)

Small Molecules Identified by an In Silico Docking Screen Targeting Anaphase-Promoting Complex/Cyclosome Subunit 1 (APC1) Potentiate Paclitaxel-Induced Breast Cancer Cell Death

Scott C. Schuyler, Rythm Gupta, Tran Thi Bao Nguyen, Cheng-Ye Weng and Hsin-Yu Chen

Supplementary Materials:

Figure S1. Validation of purity and chemical molecular identity by mass for each of the small molecules was provided upon request from the manufacturer in the form of high-performance liquid chromatography (HPLC) and mass spectrometry spectra, with each displaying a prominent single peak (Enamine Ltd., Ukraine). **A)** $C_{21}H_{19}N_3O_4$ (ZINC000005182504) “408”. **B)** $C_{17}H_{12}F_3N_3O_2$ (ZINC000014197366) “558”. **C)** $C_{19}H_{15}F_3N_4$ (ZINC000008038860) “734”. **D)** $C_{23}H_{19}F_2N_5O_2$ (ZINC000057991268) “164”. **E)** $C_{18}H_{17}N_3O_2S$ (ZINC000069037288) “686”.

Figure S2. The top 5 candidate molecules dissolved in 100% DMSO at a final molar concentration of 50 mM as stock solutions.

Figure S1A

MaxPeak: 91.55%
Ret_Time: 1.080 min

T5616827

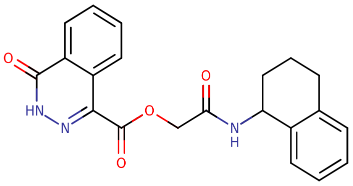

Mol Wt 377.39

Exact Mass 377.15

#	Time	Area%
1	0.563	8.45
2	1.080	91.55

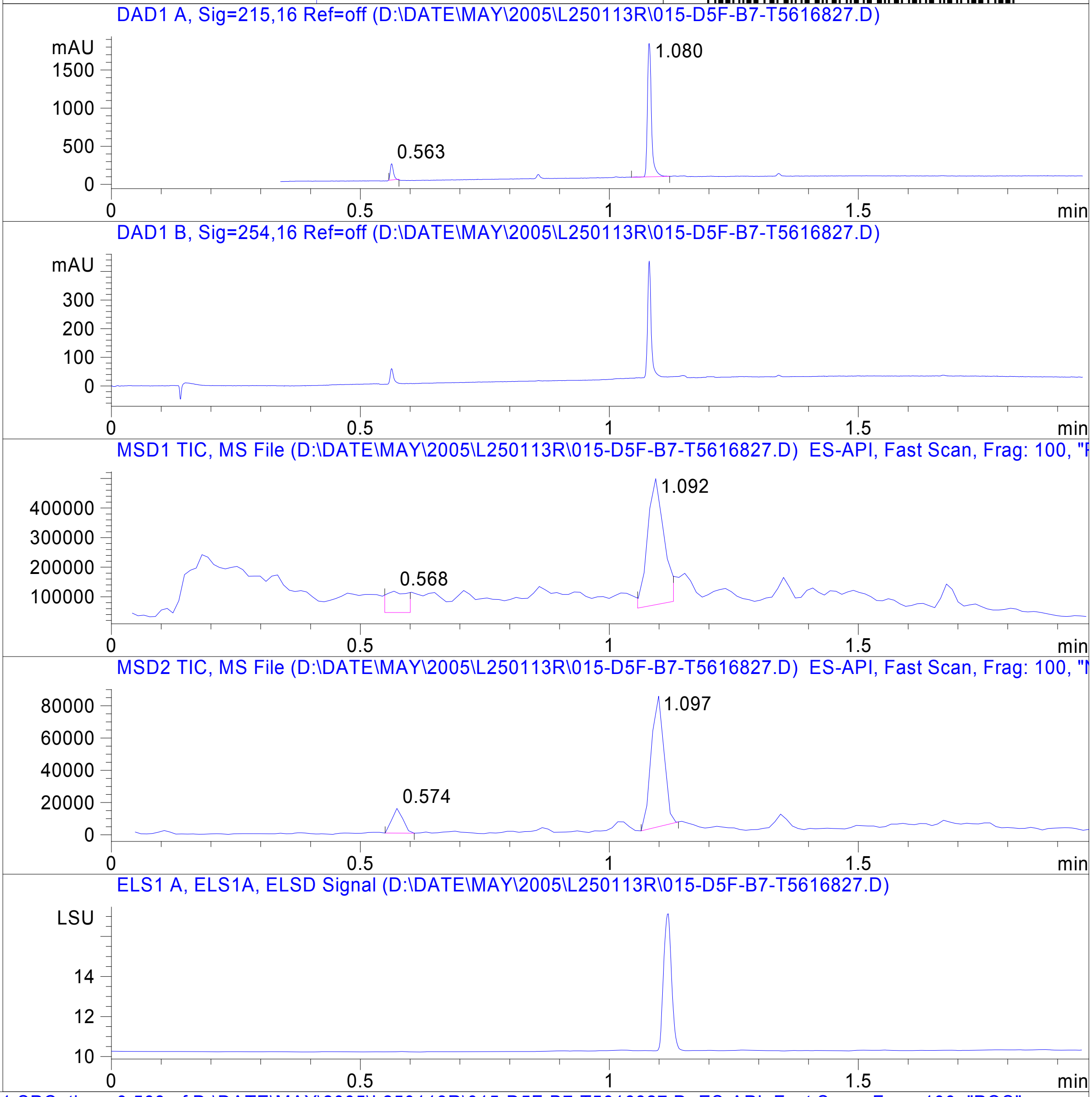

RT 0.568

RT 1.092

RT 0.574

RT 1.097

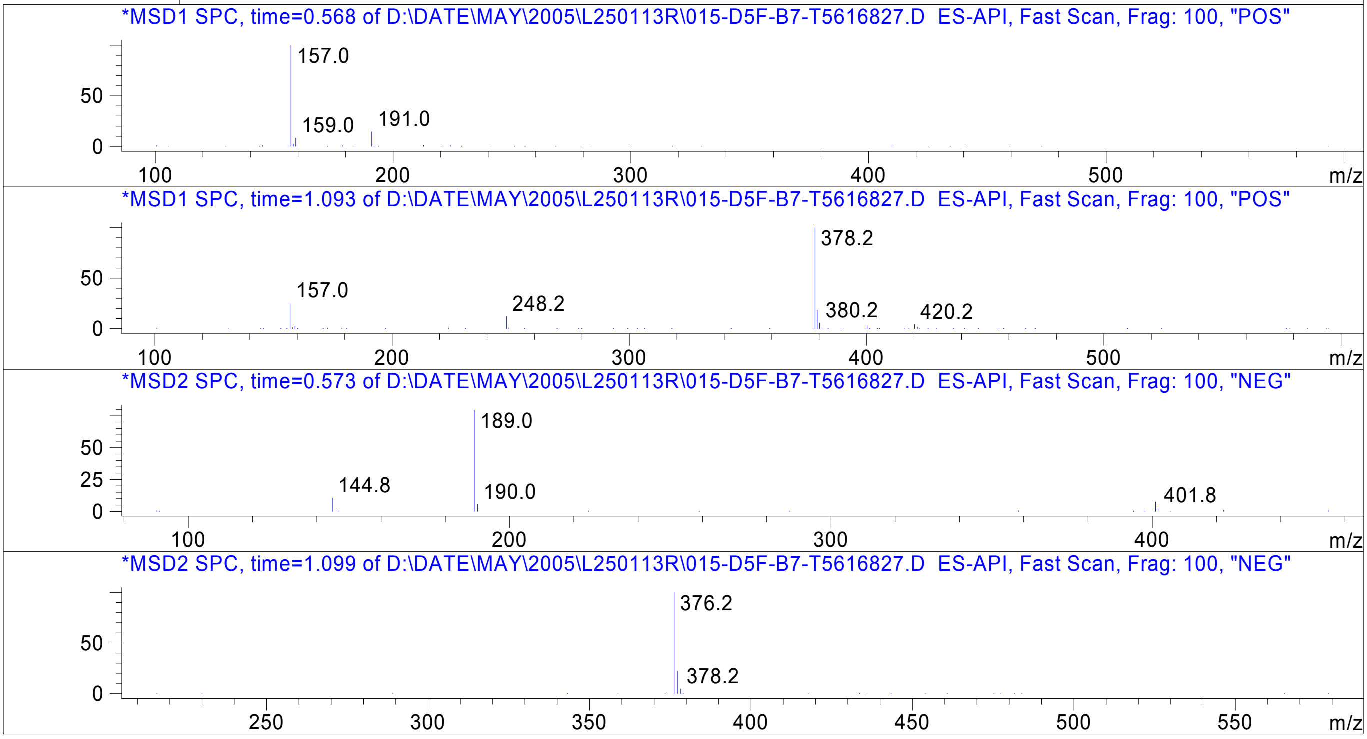

Inj.Date 5/20/2020 Y Acq. Method C:\Users\ -> ->

Figure S1B

T5863177

MaxPeak: 100.00%
Ret_Time: 1.485 min

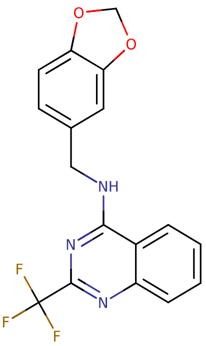

Mol Wt 347.29

Exact Mass 347.1

#	Time	Area%
1	1.485	100.00

RT 1.507

RT 1.495

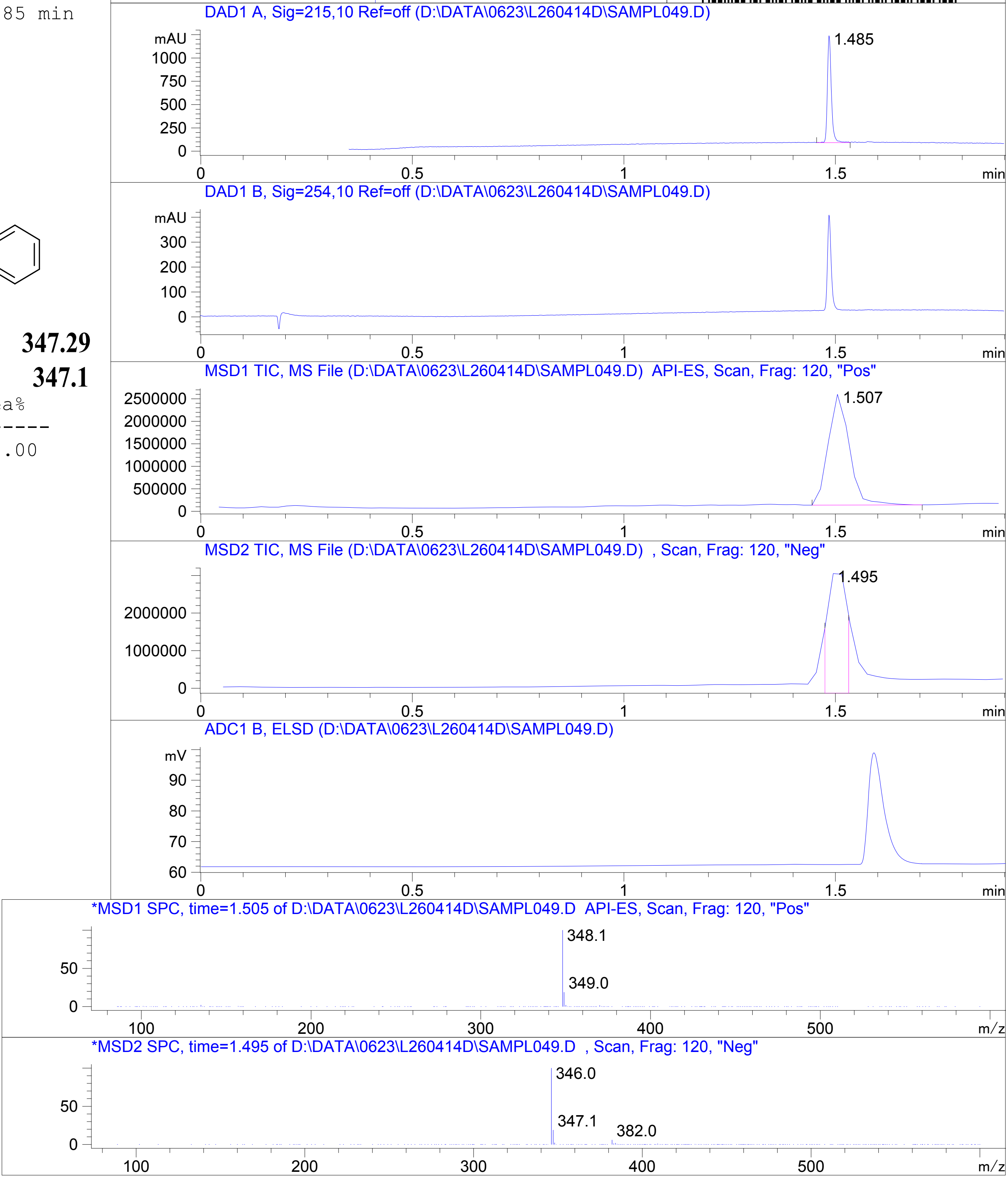

Inj.Date 6/23/2020 OA -SL- Acq. Method C:\HPCHEM\-> ->

Figure S1C

MaxPeak: 100.00%
Ret_Time: 1.548 min

T12372638$4

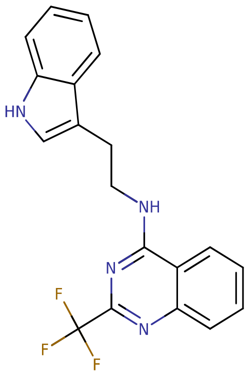

Mol Wt 356.34

Exact Mass 356.15

#	Time	Area%
1	1.548	100.00

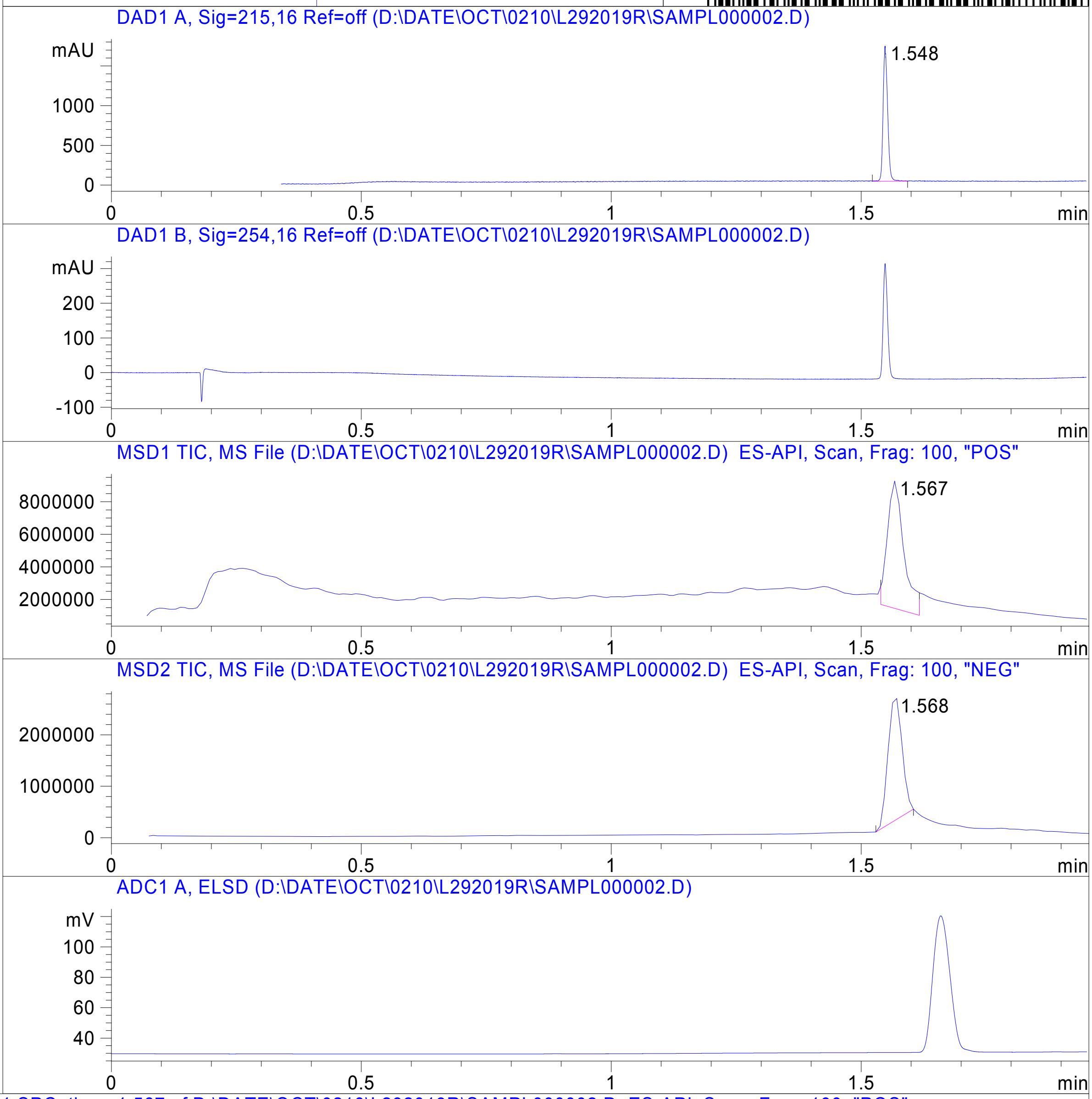

RT 1.567

RT 1.568

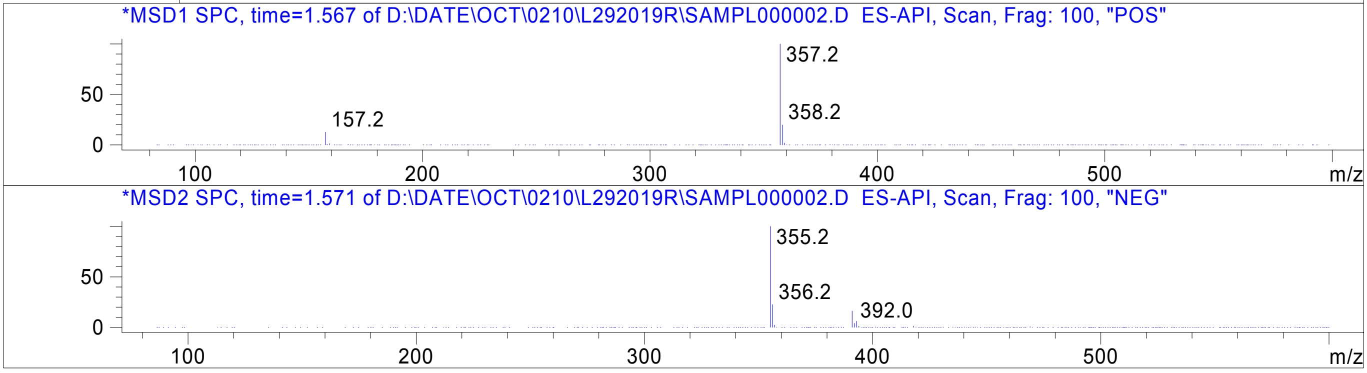

Figure S1D

```
MaxPeak:  96.84%
Ret_Time: 1.493 min
```

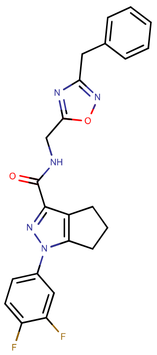

Mol Wt 435.43

Exact Mass 435.17

```
#  Time    Area%
------------------
1  1.493   96.84
2  1.527    3.16
```

T6680725

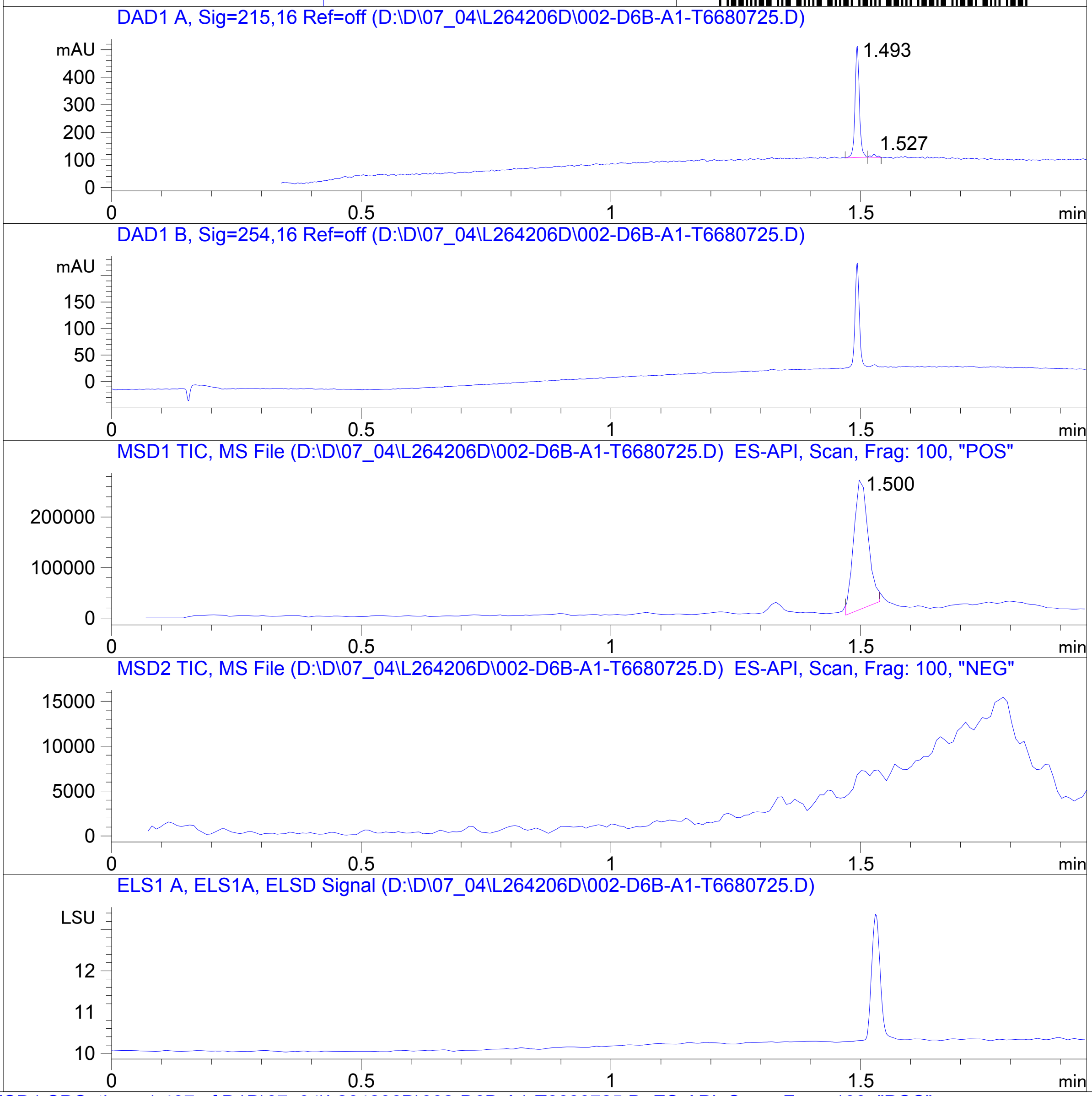

RT 1.500

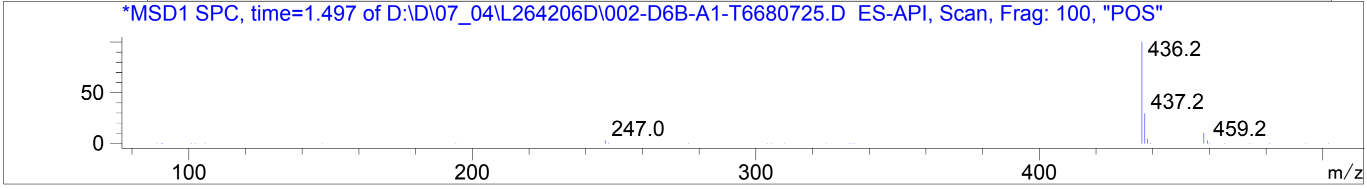

Figure S1E

MaxPeak: 100.00%
Ret_Time: 1.454 min

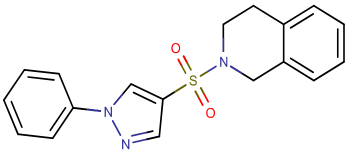

Mol Wt 339.41
Exact Mass 339.12

#	Time	Area%
1	1.454	100.00

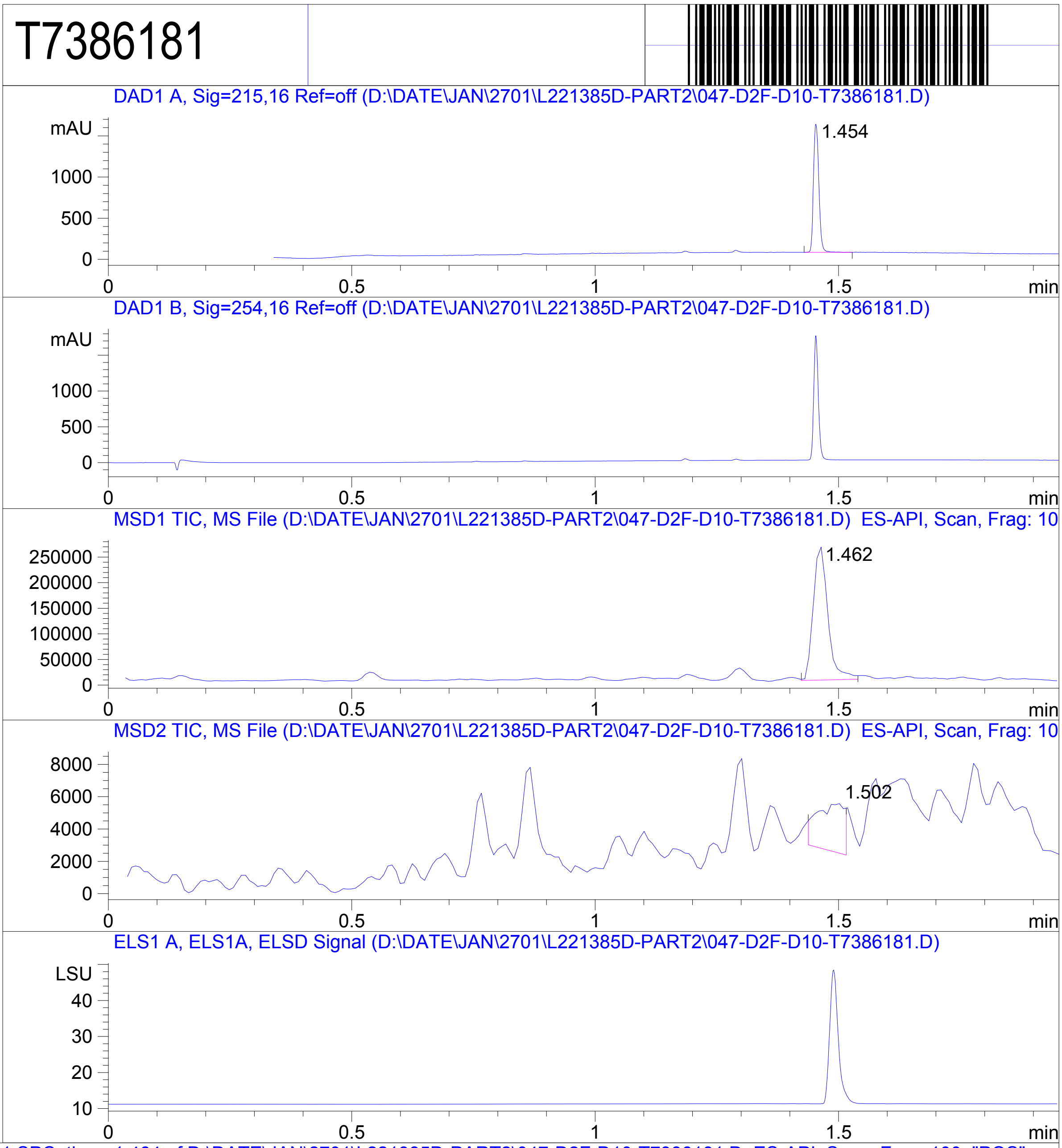

RT 1.462

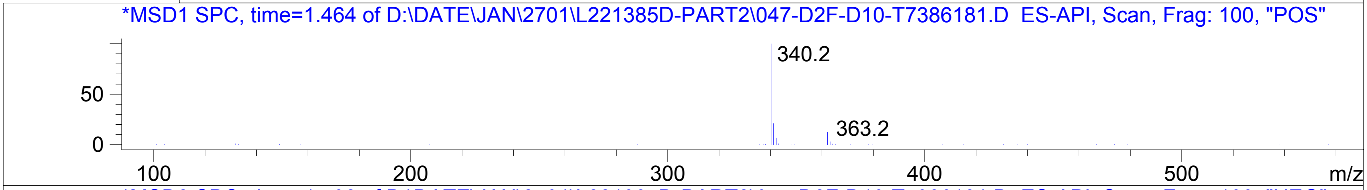

RT 1.502

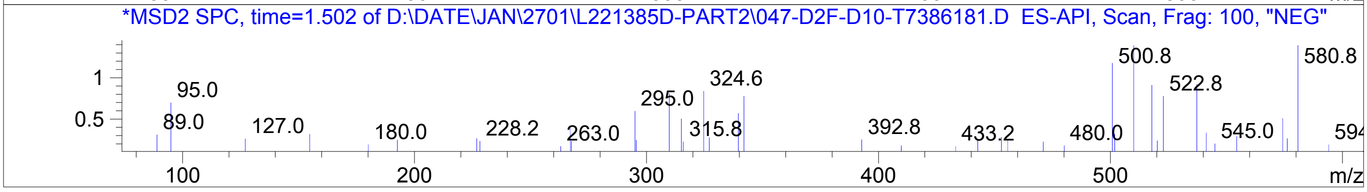

Inj.Date 1/27/2020 Y <invalid> -16- Acq. Method C:\Chem32\-> ->

Figure S2

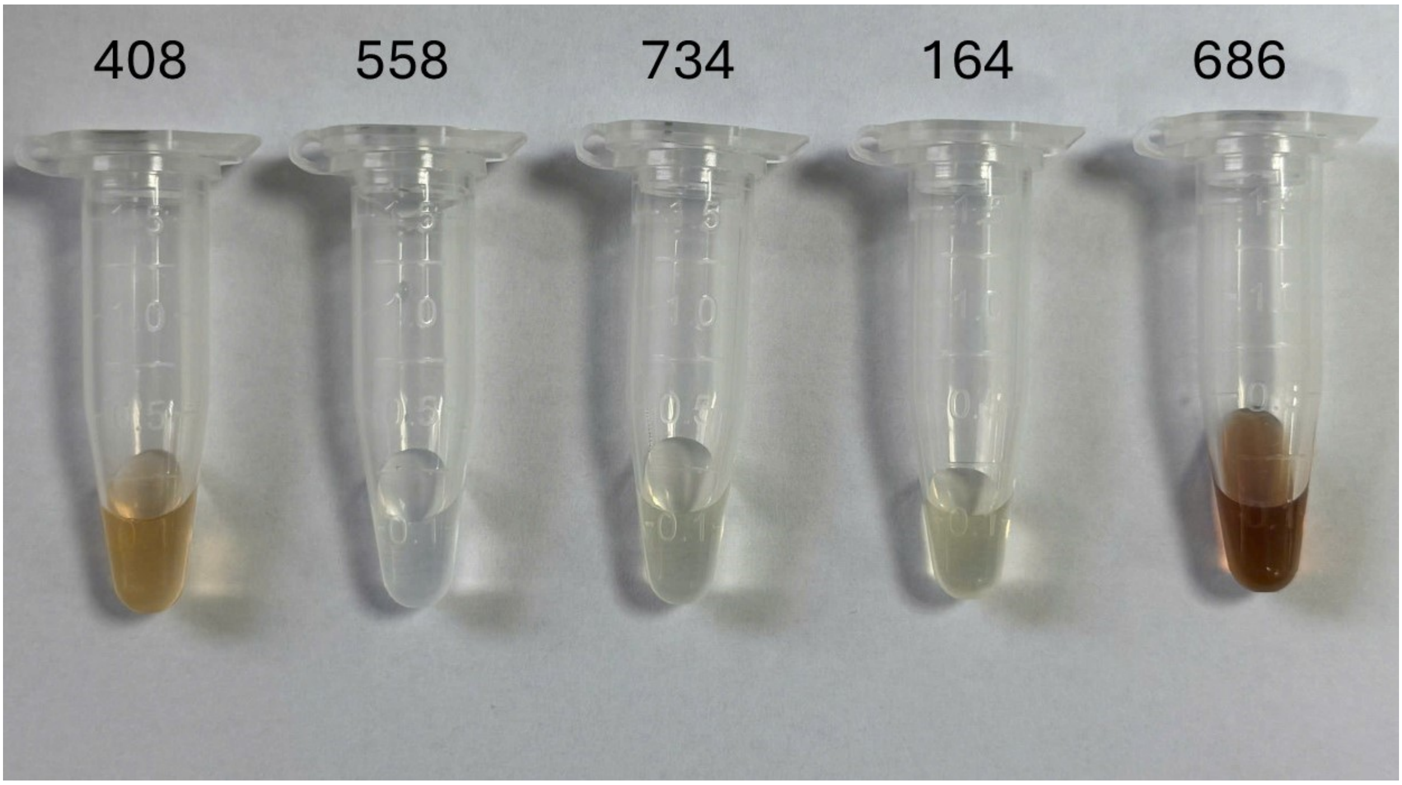

Supplement: Supplementary file 1 [file molecules-30-00895-s001.zip › molecules-3331311-supplementary.pdf]
